# Galectin-8 promotes migration and proliferation and prevents apoptosis in U87 glioblastoma cells

**DOI:** 10.1186/s40659-016-0091-6

**Published:** 2016-07-27

**Authors:** Claudia Metz, Remziye Döger, Elizabeth Riquelme, Priscilla Cortés, Christopher Holmes, Ronan Shaughnessy, Claudia Oyanadel, Catalina Grabowski, Alfonso González, Andrea Soza

**Affiliations:** 1 Departamento de Inmunología Clínica y Reumatología, Facultad de Medicina, Pontificia Universidad Católica de Chile, Av. Libertador Bernardo O’Higgins 340, 8331010 Santiago, Chile; 2 Centro de Envejecimiento y Regeneración, Departamento de Biología Celular y Molecular, Facultad de Ciencias Biológicas, Pontificia Universidad Católica de Chile, Av. Libertador Bernardo O’Higgins 340, 8331010 Santiago, Chile; 3 Fundación Ciencia y Vida, Av. Zañartu 1482, 77803444 Santiago, Chile

**Keywords:** Galectin-8, Glioblastoma, Cancer, Cell cycle, Apoptosis, Proliferation, Migration

## Abstract

**Background:**

Glioblastoma is one of the most aggressive cancers of the brain. Malignant traits of glioblastoma cells include elevated migration, proliferation and survival capabilities. Galectins are unconventionally secreted glycan-binding proteins that modulate processes of cell adhesion, migration, proliferation and apoptosis by interacting with beta-galactosides of cell surface glycoproteins and the extracellular matrix. Galectin-8 is one of the galectins highly expressed in glioblastoma cells. It has a unique selectivity for terminally sialylated glycans recently found enhanced in these highly malignant cells. A previous study in glioblastoma cell lines reported that Gal-8 coating a plastic surface stimulates two-dimensional motility. Because in other cells Gal-8 arrests proliferation and induces apoptosis, here we extend its study by analyzing all of these processes in a U87 glioblastoma cell model.

**Methods:**

We used immunoblot and RT-PCR for Gal-8 expression analysis, recombinant Gal-8 produced in a bacteria system for Gal-8 treatment of the cells, and shRNA in lentivirus transduction for Gal-8 silencing. Cell migration as assessed in transwell filters. Cell proliferation, cell cycle and apoptosis were analyzed by FACS.

**Results:**

Gal-8 as a soluble stimulus triggered chemotactic migration of U87 cells across the polycarbonate filter of transwell chambers, almost as intensively as fetal bovine serum. Unexpectedly, Gal-8 also enhanced U87 cell growth. Co-incubation of Gal-8 with lactose, which blocks galectin–glycan interactions, abrogated both effects. Immunoblot showed Gal-8 in conditioned media reflecting its secretion. U87 cells transduced with silencing shRNA in a lentiviral vector expressed and secreted 30–40 % of their normal Gal-8 levels. These cells maintained their migratory capabilities, but decreased their proliferation rate and underwent higher levels of apoptosis, as revealed by flow cytometry analysis of cell cycle, CFSE and activated caspase-3 staining. Proliferation seemed to be more sensitive than migration to Gal-8 expression levels.

**Conclusions:**

Gal-8, either secreted or exogenously enriched in the media, and acting through extracellular glycan interactions, constitutes a strong stimulus of directional migration in glioblastoma U87 cells and for the first time emerges as a factor that promotes proliferation and prevents apoptosis in cancerous cells. These properties could potentially contribute to the exaggerated malignancy of glioblastoma cells.

## Background

Glioblastoma multiform (GBM) corresponds to the highest grade (IV) and the most aggressive in clinical course among the gliomas, which account for 80 % of primary brain tumors [1, 2]. Glioblastoma cells have abnormal regulation of growth factor and integrin-mediated signaling pathways, reflected in exaggerated proliferation and migration/invasion capabilities, as well as in improved survival, avoiding apoptosis under the noxious conditions of tumor microenvironment (hypoxia and low nutrients) [1, 3–6]. The dismal prognostic of GBM has prompted intense research on the molecular basis sustaining these malignant traits. Indeed, major advances derive from genetic profiles reported by The Cancer Genome Atlas Research Network, which revealed recurrent alterations in retinoblastoma signaling, tumor protein 53 and receptor tyrosine kinase signaling and are continuously being extended to other pathways [5, 7]. However, additional approaches based on experimental analysis are also required [5]. This includes the characterization of GBM cell lines attempting to identify factors that underlie their exaggerated proliferation, survival and migration patterns in vitro, to then guide in vivo experiments.

Galectins constitute a family of 15 glycan-binding proteins that have been emerging as important modulators of a variety of cellular processes relevant to cancer biology, including proliferation, survival, apoptosis, migration and invasion [8–10]. Angiogenesis, tumor immune escape and metastasis can compromise the function of these lectins [10, 11]. Most galectin actions are exerted once secreted by some yet unknown unconventional mechanism and involve interactions of their conserved carbohydrate-recognition domains (CRDs) with beta-galactosides contained in the glycans of cell surface and extracellular matrix (ECM) components [8–10]. Variations in glycan structural complexities determine variations in the selectivity of different galectins for particular beta-galactoside-containing glycans [8]. As a consequence, galectins can play redundant or complementary functions [10]. Predominant galectin-interacting glycoproteins include integrins at the cell surface and laminin and fibronectin in the ECM, crucial in cell adhesion and migration processes [10, 12]. As modulators of cell–matrix interactions galectins can enhance or inhibit cellular adhesion depending on the biological and physiological context [11].

Galectins 1, -2, -4, -7, -8 and -9 are expressed in human brain [13] and Galectin-1, Galectin-3 (Gal-3) and Galectin-8 (Gal-8) are the most abundantly expressed in human glioma cell lines [14]. The prototype Gal-1 and the chimeric type Gal-3 have been the most studied galectins in gliomas and glioblastoma cell lines [12]. The expression levels of both Gal-1 [15–18] and Gal-3 [19] have been correlated with the more malignant glioma tumor grades, although not always for Gal-3 [12, 20, 21]. The pathogenic roles of these galectins seems to be mainly linked to cell migration and invasion, but also to chemoresistance [12], and at least Gal-3 can promote glioblastoma cells survival under stressing conditions [6]. The potential roles of other galectins expressed in the brain, specially Gal-8 found in rat and human brain [13, 22], remains little explored and even uncertain in cell migration [17], and totally unknown regarding proliferation, apoptosis and survival of glioblastoma cells [12].

Gal-8 is a tandem-repeat galectin consisting of one N- and one C-terminal CRD of different glycan selectivity. These N- and C-CRDs are spaced by a linker peptide that depending on its length gives rise to short and long Gal-8 isoforms. Gal-8 is unique among other galectins due to the particular preference of its N-terminal CRD for α2-3-sialylated glycans, implying distinct functional consequences [11, 23]. Such property makes Gal-8 especially attractive for studies on glioblastoma cells, considering the recent evidence of an increased sialylated glycans in these cells [24]. Gal-8 interacts with selective β1-integrins and with fibronectin, sharing properties with matricellular proteins [25–28]. As a matrix protein, Gal-8 promotes cell adhesion by triggering integrin-mediated signaling and cytoskeletal rearrangements [25, 26, 29], which can result in increased [25, 26] or arrested cell migration [29] depending on the cell type. Gal-8 can also induce cell arrest and apoptosis, as reported when it is used as soluble stimulus [27, 30]. Gal-8 has certainly the potential to contribute to cancer pathogenesis in different ways. Its expression increases in a variety of cancers [11], it can stimulate angiogenesis [31, 32] and might promote integrin-mediated metastasis [33]. Gal-8 expression has not been found associated with malignancy in gliomas, thus contrasting with Gal-1 and Gal-3 [17]. However, Gal-8 has been described to share with Gal-1 and Gal-3 a predominant distribution towards the invasive parts of xenografted glioblastomas and, to a lesser extent, also shares the stimulation of glioblastoma cell migration when used as a matrix protein in a two dimensional in vitro assay [17]. Here we extend the evidence showing a strong role of Gal-8 in glioblastoma cell migration, using it as a soluble stimulus on a 3D transwell assay. We also describe for the first time that Gal-8 contributes to the proliferation and anti-apoptotic/survival properties of the highly malignant glioblastoma cells.

## Methods

### Cell lines, antibodies, reagents and plasmids

U87 glioblastoma cells (ATCC) were maintained in Dulbecco’s Modified Eagle’s Medium (DMEM—4.5 g/l glucose) supplemented with 10 % fetal bovine serum (FBS), 100 Units/ml penicillin, 0.1 mg/ml streptomycin (Sigma-Aldrich, ST Louis, MO) and 5 μg/ml plasmocin. β-actin C4 monoclonal (Santa Cruz, Palo Alto, CA, USA) and cleaved-caspase-3 rabbit polyclonal antibodies (Cell Signaling Technology) were used. We generated recombinant human Gal-8 and anti-Gal-8 rabbit polyclonal antibodies as described [29, 34]. Lentiviral vectors and the viral delivery system for pVSVG, pΔR, and pLKO.1-puro vectors, the CellTrace CFSE kit for the cell proliferation assay and Alexa 488-conjugated secondary antibody were obtained from (Thermo Fisher Scientific, Waltham, MA, USA). Costar Transwell chambers with an 8 μm pore polycarbonate membrane and 6.5 mm insert were from Corning Incorporated, NY, USA.

### Viral particle production and transduction

Recombinant lentiviruses were produced by cotransfection of HEK293T cells with pVSVG, pΔR, and pLKO. 1-puro vectors containing Gal-8-specific short hairpin RNA (shGal-8) or irrelevant short hairpin RNA (shC) as control. The recombinant virus-containing media was harvested 24 and 48 h post transfection and used for transduction. U87 cells (125,000 cells/well in 6-well plates) were grown for 24 h, incubated for 20–24 h with recombinant virus-containing medium, and afterwards selected using puromycin (1 μg/ml). The cells were then amplified in T25 plastic dishes and used within 7 days. The entire procedure was repeated for each experiment.

### Cell growth assessment with crystal violet assay

Cells were seeded in 96-well plates (1500 cells/well), grown for 24 h and incubated for 4 h without serum before each treatment. Cell growth was estimated by staining the cells with crystal violet as described [29], and measuring the 570 nm absorbance, which is proportional to cell number [35].

### Cell proliferation analysis by flow cytometry

Cell proliferation was assessed with the CellTrace CFSE kit that labels free amino groups with the fluorescent CFSE die and its dilution after each cell division is analyzed by flow cytometry [36]. Cells seeded at 200,000 cells/well in a 6-well plate were incubated after 24 h with 5 μM CFSE in PBS at 37 °C for 15 min. Then the cells were grown in culture medium for 24 h and CFSE fluorescence was analyzed using a FACS Verse flow cytometer (BD Bioscience) using 488 excitation and emission filters. Fluorescence was compared with a T0 time point corresponding to cells incubated with CFSE and immediately analyzed.

### Cell apoptosis

Apoptotic cells were analyzed by both flow cytometry and fluorescence microscopy. For flow cytometry, indirect immunofluorescence was performed following the standard protocol for intracellular antigens using the cleaved caspase-3 antibody and Alexa 488-conjugated secondary antibody. For fluorescent microscopy, the cells were grown on coverslips, treated for indirect immunofluorescence with the same antibodies and the apoptotic cells were identified by the pattern of caspase-3 activation and apoptotic nuclei stained with Hoechst, as described [30].

### Migration assays in transwell filters

U87 cells deprived of serum for 4 h were seeded (50,000 cells) over 8 μm polycarbonate filters of the upper compartment of Transwell chambers in DMEM without serum. Different conditions were tested comparing the effects of DMEM alone (control) or supplemented with serum or Gal-8 added to the lower chamber for 16 h at 37 °C, as described for assaying chemoattractants [37]. The cells were stained with 0.2 % crystal violet/50 % ethanol for 10 min. Cells at the top side of the upper chamber were removed to eliminate background and those that migrated to the bottom side of the membrane were photographed and counted.

### Semiquantitative RT-PCR

Total RNA was purified with TRIzol (Invitrogen) and analyzed by RT-PCR as described [30], using the following primers: 5′-TGACCCAGATCATGTTTGGAG-3′ (sense) and 5′-TTCTCCTTAATGTCACGCAC-3′ (antisense) for β-actin; 5′-ATACTCTGCTCTATGGCCAC-3′ (sense) and 5′-TGGCATTTGCATTCACTTCT-3′ (antisense) for Gal-8.

### Quantitative RT-PCR (qRT-PCR)

The quantification of short and long isoforms of Gal-8 versus β-actin mRNAs was assessed by quantitative PCR (qPCR) in a Rotor-Gene Q from Qiagen, using the Rotor-Gene™ SYBR®Green PCR Kit (Qiagen) and the following primers: CCAGCTTAGGCTGCCATTC (Gal-8S f); AGGCGTGGGTTCAAGTGTAG (Gal-8S r); CCCAGCTTCCTAGTAATAGAGG (Gal-8L f); CTTTAACGACGACAGTTCGTC (Gal-8L r), TGACCCAGATCATGTTTGAG (human β-actin f); TTCTCCTTAATGTCACGCAC (human β-actin r). The amplification protocol was that recommended for the Quiagen Kit.

### Cell cycle analysis

Cell cycle was analyzed by flow cytometry (BD FACSverse Instrument) using the data acquisition BD FACsuite software. Briefly, subsequent to transfections, cells were trypsinized, collected into eppendorf tubes, washed with PBS by centrifugation at 700*g*, and fixed in 70 % ethanol at −20 °C for at least 2 h, and stored at −20 °C until analysis. Cells were then washed again with PBS and stained with Propidium Iodide (PI)/RNase Staining Buffer solution (BD Pharmingen) at room temperature in the dark, and PI fluorescence was analyzed by flow cytometry (protocol as described by BD Biosciences).

### Gal-8 detection

Pull-down of Gal-8 from 72 h conditioned medium of U87 cells was used to detect secreted Gal-8. Conditioned medium (4 ml) was centrifuged for 10 min at 1000*g* to eliminate cell debris and incubated with 100 μl of α-lactose-agarose beads for 3 h at 4 °C, in the presence of protease inhibitors (2 μg/ml leupeptin, 2 μg/ml pepstatin and 2 mM PMSF). Then, the beads were sedimented by centrifugation on 1000 rpm for 3 min, washed tree times with PBS and subjected to 10 % SDS-PAGE followed by immunoblot with anti-Gal-8 (1:500), generated in the laboratory, and a horseradish peroxidase-coupled rabbit IgG secondary antibody (Rockland), and developed with the enhanced chemiluminescence (ECL) method (Wester Nova 2011) as described [29, 34]. Similarly, immunoblot was used to assess Gal-8 protein levels in cells using 10 μg of total protein lysate. The intensity of the bands was quantified using the G:Box gene tools software detection system (Syngene). Quantifications are relative to actin bands used as a loading control.

### Statistics

Comparisons were statistically analyzed with the two-tailed non-paired Student’s *t* test.

## Results

The human U87 cell line expresses Gal-8 and therefore provides a useful model system to study the role of this lectin in the proliferation and migration properties of highly malignant glioblastoma cells.

### Gal-8 silencing

**Fig. 1:**
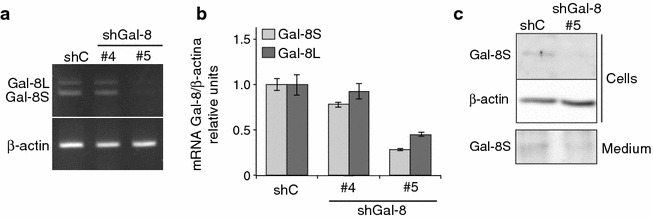
Gal-8 silencing in U87 glioma cells. U87 cells were transduced with lentiviral particles containing Gal-8-silencing shRNAs (shGal-8#4 or shGal-8#5) or irrelevant shRNA (shC). Expression of Gal-8 isoforms (Gal-8S and Gal-8L) was determined 3 days post-infection by semi-quantitative RT-PCR (**a**) qRT-PCR corrected by β-actin mRNA level, (**b**) and western blot in cell extracts (Cells) and 72 h conditioned medium (Medium) (**c**)

To study the role of endogenous Gal-8 in migration and proliferation processes of U87 cells, we first analyzed the effect of silencing its expression with shRNA. We tested the silencing potential of two different Gal-8 shRNAs (shGal-8#4 and #5) compared with an irrelevant shRNA (shC) transduced in lentiviral particles. After 3 days post-infection we assessed the expression of Gal-8S and Gal-8L isoforms by semi-quantitative RT-PCR (Fig. 1a), qRT-PCR (Fig. 1b) and western blot with a polyclonal antibody generated in our laboratory (Fig. 1c). hGal-8#5 silenced around 60 % of both Gal-8S and Gal-8L transcripts, whereas neither shC nor shGal-8#4 had detectable effects (Fig. 1a, b). At the protein level, we observed about a 60–70 % reduction of Gal-8S protein mass, both in the cells and medium (Fig. 1c). Detection of Gal-8 in the medium indicates that it is secreted, as previously described for Gal-8 and other galectins [11]. We could not detect the long isoform by western blot, presumably because the longest linker in Gal-8L includes a thrombin cleavage site that promotes protein instability [38, 39].

### Role of Gal-8 in U87 cell migration

**Fig. 2:**
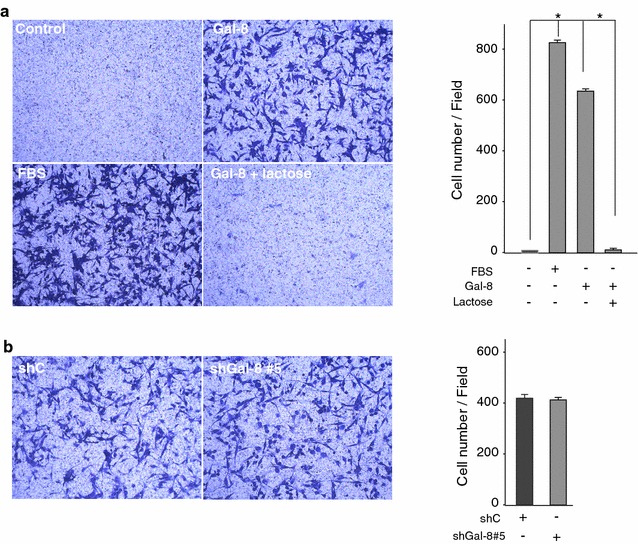
Soluble Gal-8 as a chemoattractant induces migration of U87 cells, while partial silencing of Gal-8 does not inhibit migration. **a** U87 cells (50,000 per transwell chamber) were seeded in medium without FBS in the upper compartment of transwell chambers and the lower chamber was filled with medium in the following conditions: without serum (control), 10 % FBS or 50 μg/ml Gal-8 in the absence or presence of 20 mM lactose for 16 h. The bottom sides of the filters were stained with* crystal violet* to count the cells that migrated across the filter. The* graph* represents cells per field (five different fields in duplicate for each condition) (*p < 0.0001). **b** Cell migration of U87 cells transduced by control shC or Gal-8-silencing sh-Gal-8#5, using 10 % FBS as a chemoattractant, show no significant differences

Gal-8 used as a matrix protein, pre-coating a plastic surface, has been described to stimulate motility of U373 glioblastoma cells, although to a lesser extent than Gal-1 and Gal-3 [17]. The assay measured the extent of movement based on the cells’ original position, but did not inform about movement directionality required for effective migration [17]. Here we analyzed the effect of Gal-8S as a soluble stimulus on three-dimensional (3D) cell migration across a polycarbonate membrane of Transwell chambers. We seeded the cells in the upper chamber and added medium without serum (control), with serum (FBS) or with Gal-8S in the lower chamber. This setting is widely used to test potential chemoattractants [37], as serum added to the upper chamber together with cells does not stimulate migration across the filter (not shown). After 16 h, we stained the filters with crystal violet and counted the cells that migrated from the upper to the lower side of the filter. Gal-8S strongly stimulated migration, similar to fetal bovine serum (FBS) (Fig. 2a). Co-incubation of cells with Gal-8S and 20 mM lactose, used to block the interactions of galectins with cell surface glycans [40], completely abrogated the effect of Gal-8S on U87 cell migration. These results clearly indicate that Gal-8S acting as soluble stimulus promotes migration of U87 cells.

In contrast, we did not observe differences in the migration activity of cells transduced with shGal-8#5 compared with shC (Fig. 2b). As Gal-8 protein is not completely silenced, these results suggest that the remaining Gal-8 protein (60 %) is enough to maintain cell migration.

### Role of Gal-8 on U87 cell proliferation

**Fig. 3:**
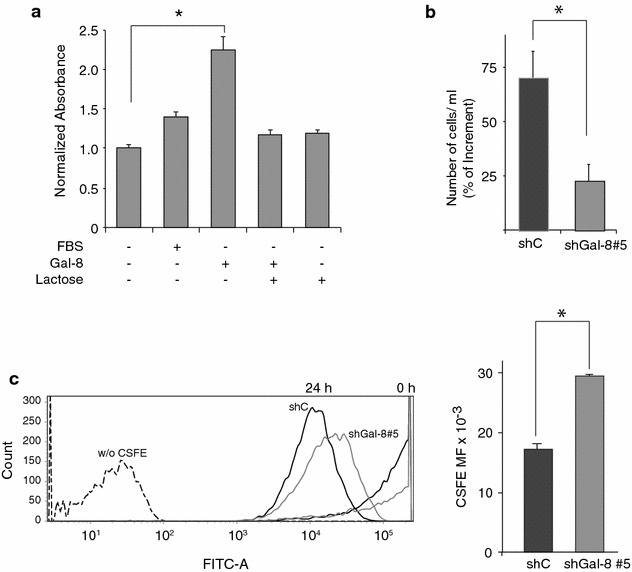
Soluble Gal-8S promotes cell growth and its partial silencing reduces proliferation of U87 cells. **a** Cell growth under Gal-8 treatment. U87 cells were seeded on plastic plates, treated with soluble Gal-8S (50 μg/ml) for 24 h in the presence or absence of 20 mM lactose and a* crystal violet* assay was performed in triplicate. Gal-8 increased cell growth (*p < 0.0001) except when co-incubated with lactose, indicating a glycan interaction-mediated effect. **b** Effect of Gal-8 silencing on cell growth. U87 cells transduced with shGal-8#5 or irrelevant shRNA (shC) lentiviral particles were cultivated for 1 week, seeded (100,000 viable cells per well) for 48 h and viable cells were counted after trypan blue staining. *Graph* represents the percentage of cell number increment in two independent experiments in triplicate (*p < 0.01). **c** Effect of Gal-8 silencing on proliferation. Transduced U87 cells were stained with CFSE and fluorescence dilution, reflecting cell proliferation, was analyzed after 24 h by flow cytometry. A representative histogram shows a lower decrease of CFSE fluorescence in shGal-8#5 versus control shC-transduced U87 cells. The* graph* represents the quantification of CFSE median fluorescence index of three independent experiments. (*p < 0.05)

In addition, we studied the cell proliferation effects of recombinant Gal-8S exogenously added to the medium. Gal-8S used as a soluble stimulus increased the proliferation of U87 cells by 1.5-fold, except when co-incubated with lactose, as shown by crystal violet staining (Fig. 3a). These results indicate that Gal-8 acting extracellularly on cell surface glycans promotes U87 cell proliferation.

We also evaluated the effect of Gal-8 silencing on U87 cell proliferation counting viable cells stained with trypan blue. Two days after plating 100,000 cells, we found that U87 cells transduced with an shC increased their cell number by 70 %, whereas U87 cells transduced with shGal-8#5 only increased their number by 10 % (student test; *p < 0.05) (Fig. 3b). These results contrast with those we previously showed on cell migration (see Fig. 2b), suggesting that the proliferation process is more sensitive to Gal-8 depletion.

**Fig. 4:**
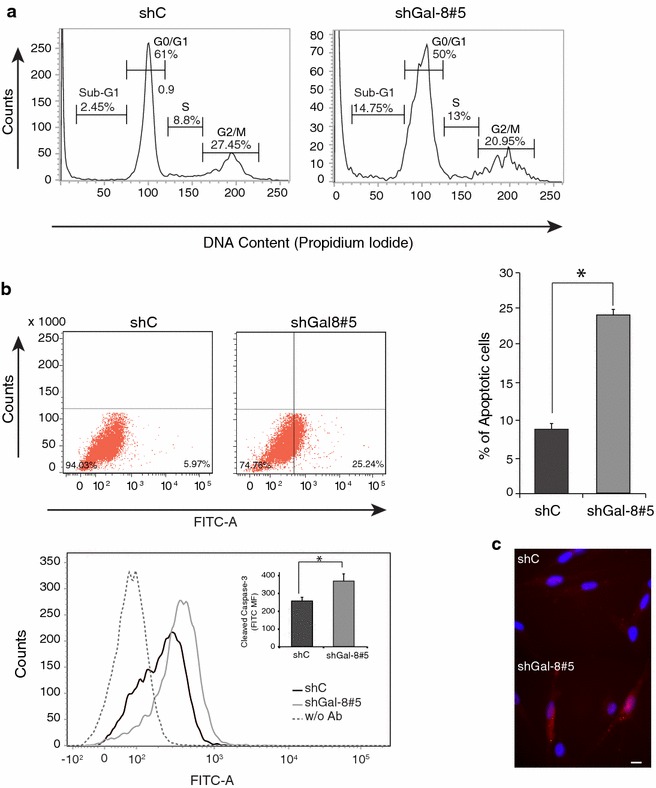
Gal-8 partial silencing increases apoptosis in U87 cells. **a** Flow cytometry analysis of cell cycle and fragmented DNA. The histogram shows the percentage of U87 cells (transduced with shC or shGal-8#5) in different phases of the cell cycle and the sub-G1 phase containing fragmented DNA, indicative of apoptotic cells. The proportion of sh-Gal-8#5-transduced cells compared with shC cells decreased in G0/G1 and G2/M, while increased in S and more significantly (sixfold) in Sub-G1. **b** Apoptosis assessed by caspase-3 activation. U87 cells transduced with shC or shGal-8#5 and treated for cleaved caspase-3 indirect immunofluorescence were analyzed by flow cytometry (**b**) a representative dot blot of two experiments with the corresponding graph and an histogram of four experiments with an* inset graph* representing normalized mean fluorescence (MF) intensity of cleaved caspase-3 reveals increased apoptosis in shGal-8-#5-transduced cells. (*p < 0.05). **c** Apoptotic cells detected by indirect immunofluorescence against cleaved caspase-3 and Hoechst stained nucleus

To define whether the effect of Gal-8 silencing on cell growth was due to reduced proliferation or increased apoptosis, we performed FACS analysis with CFSE and activated caspase-3 staining, respectively, and included a cell cycle assessment. Taking CFSE fluorescence dilution as a measure of cell proliferation, we detected an almost twofold reduction of cell proliferation in U87 cells transduced with shGal-8#5, clearly contrasting with the shC (Fig. 3c). FACS analysis of the cell cycle showed a lower proportion of U87 cells in G0/G1 under Gal-8 silencing conditions, compared to cells transduced with shC (Fig. 4a). Gal-8-silenced U87 cells also showed an increased proportion of cells in a Sub-G1 phase, which represents fractional DNA that corresponds to apoptotic cells (Fig. 4a). Flow cytometry (Fig. 4b) and indirect immunofluorescence (Fig. 4c) using antibodies against activated caspase-3 also showed higher levels of apoptosis in U87 cells silenced with the shGal-8#5. These results indicate that endogenous Gal-8 maintains high proliferation rates and protects against apoptosis in U87 cells.

## Discussion

This study complements previous observation regarding the role of galectins in glioblastoma cell migration and also shows for the first time evidence of a role of Gal-8 in promoting proliferation and preventing apoptosis of these highly malignant cells.

Migration of glioblastoma cells from the initial tumor into the surrounding brain parenchyma is a major pathogenic characteristic of diffuse GBM tumors, as it precludes an effective resection and implies post-surgical recurrence [4, 41, 42]. Glioma invasion might be a main determinant of tumor growth, as predicted by kinetics analysis [43–45]. Furthermore, migrating cells seem to be more resistant to chemotherapy [4]. Therefore, improving combat against GBM tumors requires a detailed understanding of the cellular and molecular mechanisms and the extracellular factors controlling glioma cell migration [4]. Galectins that modulate cell adhesion and migration interacting with integrins and ECM are indeed potential factors to consider in a such tumoral trait [12]. Previous studies and our present results [17, 46] indicate that Gal-8 can play complementary roles, together with Gal-1 and Gal-3, in glioma cell migration.

Camby et al. [17] reported that brain xenographs of glioblastoma cell lines H4, U373 and U87 expressed higher levels of Gal-1, Gal-3 and Gal-8 in the invasive region of the tumor. Nude mice xenographed with U87 or U373 cells expressing low levels of galectin-1 (transfected with antisense mRNA) survive longer than mice graphed with cells expressing normal levels of Gal-1 [46]. These observations suggest that galectin-mediated brain invasion contributes to the aggressiveness of these tumors. Camby et al. [17] also described that Gal-1 and Gal-3, and to a lesser extent, Gal-8, stimulate glioblastoma cell migration in vitro. The migration assay used in that study measured two-dimensional motility on a plastic surface pre-coated with galectin and did not report directionally of the movement. Instead, we used an experimental setting described for chemoattractant stimulus of cell migration across a polycarbonate filter of transwell chambers [37]. We found that Gal-8 as soluble stimuli induces a marked trans-filter migration of U87 cells, almost as intensively as FBS. Therefore, soluble Gal-8 is a very strong stimulus of directional migration in glioblastoma U87 cells.

Even though Gal-1, Gal-3 and Gal-8 increase glioblastoma cell migration, their experimental silencing has different effects. The migratory activity of A172, U118 and U87 glioma cells is reduced upon Gal-1 silencing [47]. In contrast, Gal-3 deficiency has been described to increase motility in U373 glioblastoma cells cultivated on laminin [48]. We show that silencing around 60–70 % of the Gal-8 protein does not affect the migration activity of U87 cells elicited by FBS in the chemotaxis assay. The remaining Gal-8 might be enough to maintain migration, likely complementing the roles of Gal-1 and Gal-3, endogenously expressed by U87 cells [17].

Cell migration is a complex process involving protrusion of the leading edge, adhesion to the substratum, retraction of the rear, and de-adhesion. This process involves integrin-mediated cell adhesion to ECM and signaling through focal-adhesion-kinase (FAK) and Rho-GTPases leading to actin cytoskeleton rearrangements [49–51]. Galectins can stimulate this cell motility machinery by interacting with integrins [52]. For instance, Gal-1-induced migration of glioma cells has been associated with modifications to actin cytoskeleton organization and the increase in small GTPase RhoA expression [46]. Gal-3 can induce actin re-organizations related to cell motility involving EGFR and caveolin-1-mediated activation of Rho [53]. Although we did not assess here the effects of Gal-8 on these pathways, previous studies have shown that Gal-8 used as a matrix promotes cellular attachment, spreading, and migration by engaging integrin signaling towards FAK, paxillin [26]. We described that Gal-8 activates Rho-GTPase Rac-1 leading to cell spreading and actin cytoskeletal assembly of lamellipodia in Jurkat cells [29]. Therefore, Gal-1, Gal-3 and Gal-8 have recognized potential to modulate the cytoskeletal machinery that orchestrates cell migration and can play complementary roles in this process.

In addition, we show that U87 cells respond to exogenously added recombinant Gal-8 by increasing their proliferation rate. Gal-8 silencing in conditions that reduce the mass of Gal-8 protein by 60–70 %, not only reduces proliferation but also increases the apoptotic rate of these cells. As mentioned, migratory activity of these cells remains unaffected under these conditions, suggesting that migration can be maintained at lower levels of Gal-8 than those required for proliferation. Our results indicate that endogenous Gal-8 expression sustains both proliferation and survival of these highly malignant cells.

To our knowledge, this is the first example of tumor cell proliferation requiring endogenous Gal-8 expression or stimulated by exogenous Gal-8. The only other example of Gal-8-induced proliferation is in naïve CD4(+) T cells [54]. Previous studies in several cell lines, including the human small-cell lung carcinoma cells, reported growth arrest and even apoptosis in response to Gal-8 added as a soluble stimulus or overexpressed by transfection, involving interactions with selected β1-integrins and integrin-mediated signaling [26, 27, 55]. Tribulati et al. found that Gal-8-induced apoptosis in a subpopulation of CD4(high)/CD8(high) thymocytes [56]. We described apoptosis induced by Gal-8 in Jurkat cells and CD3/CD8 activated human peripheral T cells, involving phospholipase D/phosphatidic acid signaling [30]. Cataneo et al. [57] also reported Gal-8-induced apoptosis in activated T cells. More recently, Ruiz et al. [58] showed that Gal-8 inhibits cell proliferation in neuroblastoma, erythroleukemia and colon adenocarcinoma cells. Therefore, Gal-8 has dual effects, either stimulating or inhibiting proliferation depending on the cell context.

Downregulation of Gal-1 has been shown to reduce proliferation of A172 but not U118 glioblastoma cells [47]. However, contrasting with our results in U87 cells, recombinant Gal-1 had no effect on the proliferation of A172 and U118 cells [47]. U343 cells transfected to overexpress Gal-1 increased their proliferation rates, whereas 60 % silencing of Gal-1 in U87 cells had no effect on proliferation [59]. It seems that the role of Gal-1 in proliferation depends on intracellular mechanisms whereas its role in migration is both intracellular and extracellularly exerted [47]. Gal-3 likely contributes to glioma cell growth increasing cell survival in hypoxic and nutrient-deprived tumor microenvironments [6]. In U87 cells, Gal-3 knockdown reduces tumor growth in nude mice without affecting cell proliferation in vitro [6]. Taken together with our results, these data suggest that different galectins can contribute in different ways to processes of proliferation, migration and survival in glioblastoma cells.

We found Gal-8 in conditioned media and show that lactose completely blocks the effects of Gal-8 on cell migration and proliferation, indicating that U87 cells secrete Gal-8 and this lectin modulates these processes interacting with cell surface glycans. It is interesting to remark that glioblastoma cells have been reported to display an increased expression of α2-3-sialyltransferase leading to higher terminal sialic acid on glycans [24]. Among all other galectins, a unique feature of Gal-8 is the strong affinity for α2-3-sialylated oligosaccharides residing in its N-terminal CRD [60]. Therefore, it is possible that Gal-8 plays a major role in glioblastoma malignancy, which can be specifically targeted by taking advantage of its unique glycan selectivity. Our results support this possibility revealing for the first time a role of Gal-8 in proliferation, survival and migration in a widely used glioblastoma cell model.

## Conclusions

Gal-8 has the potential to contribute to the malignancy of glioblastoma cells by promoting proliferation, survival and migration capabilities. In contrast to other cell systems, Gal-8 reduces the apoptotic rate in U87 glioblastoma cells.
